# Curcumin ((1E,6E)-1,7-bis(4-Hydroxy-3-methoxyphenyl)-1,6-heptadiene-3,5-dione) Induces Apoptosis-like Death in *Leishmania amazonensis* Promastigotes and Exhibits Leishmanicidal Activity in Infected Macrophages in Free and Beeswax-Based Nanoparticle Formulations

**DOI:** 10.3390/pathogens15060650

**Published:** 2026-06-20

**Authors:** Amanda Cristina Machado Carloto, Ana Carolina Jacob Rodrigues, Mariana Barbosa Detoni, Ellen Mayara Souza Cruz, Virgínia Márcia Concato-Lopes, Rodolfo Bento Balbinot, Fabrício Seidy Ribeiro Inoue, Yuri Barreiros, Arthur Poester Cordeiro, Pedro Henrique Hermes de Araújo, Claudia Sayer, Paulo Emílio Feuser, Celso Vataru Nakamura, Ivete Conchon-Costa, Danielle Lazarin-Bidóia, Wander Rogério Pavanelli

**Affiliations:** 1 Laboratory of Immunoparasitology of Neglected Diseases and Cancer, State University of Londrina, Londrina 86057-970, PR, Brazil; amanda.carloto@gmail.com (A.C.M.C.);; 2 Cedars-Sinai Medical Center, Cedars-Sinai, Los Angeles, CA 90048, USA; 3 Laboratory for Technological Innovation, Development of Pharmaceuticals and Cosmetics, State University of Maringá, Maringá 87020-900, PR, Brazil; 4 Department of Chemical Engineering and Food Engineering, Federal University of Santa Catarina, Florianópolis 88040-900, SC, Brazilpedro.h.araujo@ufsc.br (P.H.H.d.A.); claudia.sayer@ufsc.br (C.S.);

**Keywords:** curcumin, Leishmaniais, nanoencapsulation, polyphenol, solid lipid nanoparticle

## Abstract

Leishmaniasis is a neglected tropical disease caused by parasites of the genus *Leishmania*. Curcumin (CUR) is a polyphenol with several biological properties, including antimicrobial effects. However, its low bioavailability remains a challenge, and nanoencapsulation may represent a useful strategy to overcome this limitation. This study aimed to evaluate, in vitro, the antipromastigote activity of free CUR and the antiamastigote effect of CUR nanoparticles and their association with antimoniate, as well as to elucidate possible mechanisms of action. Free CUR directly inhibited promastigote proliferation, with an IC_50_ of 25 µM at 24 h. CUR induced mitochondrial hyperpolarization, increased the production of reactive oxygen species (ROS) and nitric oxide (NO), and enhanced lipid peroxidation and the accumulation of lipid droplets in promastigotes. These alterations were associated with autophagic and apoptotic processes, morphological and ultrastructural changes, DNA fragmentation, and cell cycle arrest. Free CUR also reduced the viability of BALB/c peritoneal macrophages, and this effect was attenuated after nanoencapsulation. Free CUR, CUR nanoparticles, and their association with antimoniate (AM) reduced both the percentage of infected macrophages and the number of intracellular amastigotes at all tested concentrations, with increased NO production observed at the highest concentrations of free CUR. Altogether, our findings suggest that CUR exerts leishmanicidal activity against promastigotes by disrupting oxidative metabolism and triggering autophagic and apoptotic pathways, while amastigote elimination appears to occur through mechanisms independent of oxidative stress.

## 1. Introduction

Leishmaniasis is a neglected vector-borne tropical disease caused by at least 20 parasite species belonging to the genus *Leishmania*. Infection is transmitted between mammalian hosts through the bite of infected female sandflies [1,2]. Among its clinical manifestations, tegumentary leishmaniasis is the most prevalent worldwide, with a broad geographic distribution across 88 countries on four continents [1,3]. It is estimated that 0.7–1.2 million new cases occur annually, predominantly in countries in Asia, Africa, and South America, including Brazil [4].

The first-line treatment for leishmaniasis relies on pentavalent antimonials, which have been in use since the 1940s [5]. Although effective, their clinical application is frequently limited by the requirement for daily parenteral administration, treatment failure, adverse effects, and severe cardiotoxicity, nephrotoxicity, and hepatotoxicity [6]. When antimonials are contraindicated, alternative drugs such as amphotericin B, liposomal amphotericin B, pentamidine, paramomycin, and miltefosine may be used [7]. However, these therapies are also associated with important limitations, including toxicity, high cost, and restricted accessibility. Therefore, the search for new and safer alternatives has become a critical public health priority.

Several studies have highlighted the therapeutic potential of medicines derived from natural products, particularly those of plant origin [8]. Curcumin (1,7-bis(hydroxy-3-methoxyphenyl)-1,6-heptadiene-3,5-dione) is the main natural polyphenol found in turmeric (*Curcuma longa*). Numerous studies have demonstrated that curcumin exhibits biological activity at the cellular level [9], including anti-inflammatory, hypoglycemic, antioxidant, and wound-healing properties. Therapeutic effects on the nervous, respiratory, cardiovascular, urinary, reproductive, digestive, musculoskeletal, endocrine, and integumentary systems have also been reported [10,11,12,13,14,15]. In addition, curcumin exhibits a broad spectrum of antimicrobial activities, including antiviral, antibacterial, antifungal, and antiparasitic effects [12,15,16]. Importantly, curcumin has shown promising activity against *Leishmania* spp., demonstrating both direct leishmanicidal effects on *L. major* promastigotes and immunomodulatory activity in infected host cells, including the upregulation of IFN-γ, TNF-α, and iNOS, as well as increased reactive oxygen species (ROS) production [17,18,19,20,21,22,23,24,25].

A major limitation associated with curcumin is its low bioavailability [26], which is attributed to rapid metabolism, the inactivity of its metabolic products, and/or rapid systemic clearance [27]. To overcome these drawbacks, drug delivery systems such as nanoparticles can be employed as an effective strategy to enhance curcumin stability and bioavailability [12]. Solid lipid nanoparticles (SLNs) are nanometric colloidal systems composed of a solid lipid core capable of encapsulating both lipophilic and hydrophilic drugs [28,29,30]. SLNs offer several advantages, including drug protection, ease of large-scale production, biocompatibility, biodegradability, and reduced toxicity compared with free drugs [31,32].

Although previous studies have demonstrated the antileishmanial activity of curcumin, the mechanisms underlying parasite death in promastigote and amastigote forms of *L. amazonensis* following treatment with free or nanoparticle-loaded curcumin remain unclear. Therefore, this study aimed to evaluate the in vitro antipromastigote activity of free curcumin and the antiamastigote activity of curcumin-loaded SLNs, alone or in combination with antimoniate, as well as to elucidate their possible mechanisms of action.

## 2. Materials and Methods

### 2.1. Curcumin (CUR)

Commercial curcumin (CUR; purity ≥ 95%) was obtained from Neon Comercial (Suzano, SP, Brazil, Reference No. 02888). CUR was solubilized in absolute ethanol according to the manufacturer’s technical specifications. The final ethanol concentration did not exceed 0.1% in any experiment.

### 2.2. Production of Curcumin-Loaded Solid Lipid Nanoparticles (SLNs)

Encapsulation was performed using a combined molten dispersion and double oil/water (O/W) emulsion technique, as described by Barreiros et al. (2025) [33]. Briefly, beeswax (450 mg) was melted at 70 °C together with soy lecithin (45 mg) and Crodamol GTCC (0.42 mL; Alpha Química, Cachoeirinha, RS, Brazil) under magnetic stirring. Subsequently, 0.4 mL of water (internal aqueous phase) was added to the lipid phase under magnetic stirring (50 rpm for 5 min), followed by ultrasonication. Next, 9 mL of an aqueous phase containing Tween 80 (Vetec, Sao Paulo, SP, Brazi; 9 mg/mL) was added to the O/W emulsion which was maintained under magnetic stirring (400 rpm for 10 min at 70 °C). The emulsion was then sonicated again at 70% amplitude for 1 min to obtain a water/oil/water (W/O/W) double emulsion. Finally, the resulting emulsion was transferred into 35 mL of cold water (7 °C) under magnetic stirring (400 rpm) for 5 min, yielding unloaded (blank) SLNs.

Using the same procedure, SLNs incorporating meglumine antimoniate (AM) or CUR were prepared. For AM-loaded SLNs, AM (300 mg/mL) was used in place of the internal aqueous phase. For CUR-loaded SLNs, a CUR solution (50 mg/mL) was incorporated into the lipid phase in place of pure Crodamol.

The SLNs were centrifuged (MiniSpin, Eppendorf, Germany) at 1000 rpm for 30 min, and the supernatant was collected and centrifuged again under the same conditions. The final pellet was resuspended in 10% (*w*/*v*) dehydrated D-(+)-trehalose, frozen at −80 °C for 48 h, and subsequently lyophilized for 48 h (L101, Liobras, São Carlos, SP, Brazil). Encapsulation efficient was previously determined by Barreiros et al. (2025) using ultrafiltration followed by UV/Vis spectroscopy at 425 nm, using a Hitachi spectrophotometer (U-1900, Hitachi High-Tech Corporation, Tokyo, Japan) [33].

### 2.3. In Vitro Leishmanicidal Activity Against L. amazonensis Promastigotes

#### 2.3.1. Maintenance of Promastigote Forms

Promastigote forms of *Leishmania* (*Leishmania*) *amazonensis* (MHOM/BR/Josefa strain) obtained from the State University of Maringá were maintained in Medium 199 (GIBCO, Invitrogen, New York, NY, USA) supplemented with 10% fetal bovine serum (FBS) (GIBCO, Invitrogen), 1 M HEPES, 1% human urine, 1% L-glutamine, penicillin-streptomycin (GIBCO, Invitrogen), and 10% sodium bicarbonate. Cultures were maintained in a B.O.D. incubator at 25 °C in 25 cm^2^ culture flasks. Stationary-phase promastigotes (5-day cultures) were used in all experiments at a concentration of 10^6^ parasites/mL, unless otherwise specified. Parasite density was determined using a Neubauer hemocytometer after formalin immobilization, and the suspension was adjusted to the desired concentration in supplemented medium [34].

#### 2.3.2. Antipromastigote Activity

The antipromastigote activity assay was performed according to Assolini et al. [34]. Promastigote forms of *L. amazonensis* were treated with different concentrations of CUR (6.25, 12.5, 25, 50, 100, and 200 µM) in 24-well plates. Parasite counts were performed using a Neubauer hemocytometer after 24 and 48 h of treatment. Untreated parasites cultured in Medium 199 were used as the negative control, and ethanol (0.1%) was used as the vehicle control. Dose–response curves were generated, and the concentration required to inhibit parasite growth by 50% (IC_50_) was calculated by nonlinear regression analysis using GraphPad Prism 5.00 (GraphPad Software, Inc., San Diego, CA, USA).

#### 2.3.3. Determination of Mitochondrial Membrane Potential (∆Ѱm)

Promastigote forms were treated with the IC_50_ of CUR (25 µM) for 24 h in 24-well plates, as described by Carloto et al. [35]. The mitochondrial membrane potential (∆Ѱm) was assessed using tetramethylrhodamine ethyl ester (TMRE; Sigma-Aldrich, St. Louis, MO, USA). Parasites were washed with PBS and incubated with 2.5 µM TMRE for 30 min at 25 °C. Fluorescence was measured using a GloMax^®^ fluorimeter (Promega, Madison, WI, USA) at excitation and emission wavelengths of 480 and 580 nm, respectively. In addition, micrographs of treated promastigotes were acquired using an EVOS^®^ FL Auto Cell Imaging System (Thermo Fisher, Multiskan GO, Waltham, MA, USA) at 200× magnification. Fluorescence intensity values were normalized to parasite counts.

#### 2.3.4. Production of Reactive Oxygen Species (ROS) in Promastigotes

ROS production was evaluated as described by Assolini et al. (2020) [34] Promastigotes were treated with 25 µM CUR for 24 h, washed with PBS, and incubated with 10 µM 2′,7′-dichlorofluorescein diacetate (H_2_DCFDA; Sigma-Aldrich) diluted in DMSO for 45 min at 25 °C in the dark. Hydrogen peroxide (H_2_O_2_; 50 µM) was added for 30 min and used as a positive control. ROS levels were quantified by measuring the fluorescence resulting from the oxidation of H_2_DCFDA to 2′,7′-dichlorofluorescein (DCF) at excitation and emission wavelengths of 488 and 530 nm, respectively, using a GloMax^®^ fluorimeter. Fluorescence values were normalized to parasite counts. Representative fluorescence micrographs were obtained at 200× magnification.

#### 2.3.5. Scanning and Transmission Electron Microscopy

Promastigotes treated with 25 µM CUR for 24 h were collected by centrifugation and washed with 0.01 M PBS (pH 7.2). For scanning electron microscopy (SEM), parasites were fixed in 2.5% glutaraldehyde in 0.1 M sodium cacodylate buffer, adhered to poly-L-lysine-coated coverslips for 12 h, post-fixed with osmium tetroxide for 1 h, dehydrated in graded ethanol solutions (30–100%), critical-point dried with CO_2_, gold coated, and analyzed using a FEI SCIOS electron microscope (Hillsboro, OR, USA).

For transmission electron microscopy (TEM), parasites were fixed in 2.5% glutaraldehyde in 0.1 M sodium cacodylate buffer, post-fixed in 1% osmium tetroxide with 0.8% potassium ferrocyanide in the dark at room temperature for 1 h, dehydrated in graded acetone (50–100%), embedded in EPON resin, and polymerized at 60 °C for 72 h. Ultrathin sections were cut using an ultramicrotome, mounted on a copper grids, and contrasted with uranyl acetate and lead citrate for 20 and 10 min, respectively. Analysis were performed using a JEOL JEM 1400 transmission electron microscope (Tokyo, Japan), according to procedures previously described by Lorenzetti et al. (2026) [36].

#### 2.3.6. Nitric Oxide (NO) Detection

Promastigotes were treated with the IC_50_ of CUR for 24 h and incubated with 1 µM DAF-FM diacetate for 30 min in the dark. Subsequently, parasites were washed, resuspended in PBS, and incubated for an additional 15 min. Fluorescence was analyzed by flow cytometry using a FACSCalibur flow cytometer (BD Biosciences, San Jose, CA, USA) equipped with CellQuest software version 5.2.1 (BD Biosciences, San Jose, CA, USA). H_2_O_2_ (50 µM) was used as a positive control. A total of 10,000 events were acquired for each sample.

#### 2.3.7. Detection of Lipid Droplets by Nile Red (NR) Staining

Promastigotes were treated with the IC_50_ of CUR (25 µM) for 24 h, washed twice with PBS and directly labeled with 10 µg/mL Nile red (NR) (Sigma-Aldrich) for 30 min at 25 °C, as described by Carloto et al. [35]. Fluorescence was measured using a GloMax^®^ fluorimeter at wavelengths of 530 nm and 635 nm for excitation and emission, respectively. Fluorescence readings were normalized to parasite counts.

#### 2.3.8. Lipid Peroxidation Assay

Lipid peroxidation was assessed using diphenyl-1-pyrenylphosphine (DPPP; Sigma-Aldrich). Promastigotes treated with the IC_50_ of CUR (25 µM) for 24 h were incubated with 50 µM DPPP for 15 min at 22 °C. Fluorescence was measured at excitation and emission wavelengths of 355 and 460 nm, respectively, using a Victor X3 microplate reader (PerkinElmer, Waltham, MA, USA). H_2_O_2_ (50 µM) was used as a positive control.

#### 2.3.9. Detection of Autophagic Vacuoles

Autophagic vacuoles were detected using monodansylcadaverine (MDC; Sigma-Aldrich). Promastigotes treated with 25 µM CUR for 24 h were incubated with MDC (5 µL) for 1 h at 25 °C and fluorescence was assessed using a GloMax^®^ fluorimeter at excitation and emission wavelengths of 380 and 525 nm, respectively. Fluorescence intensity was normalized to parasite counts.

#### 2.3.10. Phosphatidylserine Exposure

Phosphatidylserine exposure was detected using Annexin-V FITC (Invitrogen, Eugene, OR, USA). Promastigotes treated with the IC_50_ of CUR for 24 h were incubated with 5 µL of Annexin-V FITC for 15 min at 25 °C. Camptothecin (CPT; 0.5 µM) was used as a positive control. Fluorescence intensity was measured using a GloMax^®^ fluorimeter at excitation and emission wavelengths of 488 and 520 nm, respectively, and normalized to parasite counts.

#### 2.3.11. Cell Membrane Integrity

Cell membrane integrity was assessed using propidium iodide (PI; Sigma-Aldrich; 0.5 µg/mL) staining. Promastigotes treated with the IC_50_ of CUR for 24 h were incubated with PI for 15 min at 25 °C. Digitonin was used as a positive control. Fluorescence intensity was analyzed using a GloMax^®^ fluorimeter at excitation and emission wavelengths of 480 and 580 nm, respectively, and normalized to parasite counts.

#### 2.3.12. Cell Size Analysis

Promastigotes treated with the IC_50_ of CUR for 24 h were washed twice and resuspended in PBS. Cell size was determined using a FACSCalibur flow cytometer (BD Biosciences) based on forward scatter (FSC-H) analysis of 10,000 events [37]. Actinomycin D (20 mM) was used as a positive control.

#### 2.3.13. DNA Fragmentation

DNA double-strand breaks were evaluated using the TUNEL assay. Promastigotes were treated with the IC_50_ of CUR for 24 h, then fixed and processed according to the manufacturer’s instructions. Actinomycin D (20 mM) was used as a positive control. The data acquisition and analysis were performed using a FACSCalibur flow cytometer (BD Biosciences) equipped with CellQuest software. A total of 10,000 events were acquired in the region corresponding to the parasites [37].

#### 2.3.14. Cell Cycle Analysis

Promastigotes treated with the IC_50_ of CUR for 24 h were fixed in 70% cold methanol in PBS at 4 °C for 2 h. Subsequently, parasites were washed with PBS, and propidium iodide (PI) and RNAse A (Sigma-Aldrich, USA) were added at a final concentration of 10 µg/mL. Samples were incubated at 37 °C for 45 min. The data acquisition and analysis were performed using a FACSCalibur flow cytometer (BD Biosciences). Taxol (10 µM) was used as a positive control. A total of 10,000 events were acquired in the region that corresponded to the parasites. The percentages of cells in each phase of the cell cycle were determined based on PI fluorescence [38].

### 2.4. In Vitro Leishmanicidal Activity in Infected Macrophages

#### 2.4.1. Experimental Animals

BALB/c mice were used as a source of primary peritoneal macrophages, the main host cells for *Leishmania* spp., allowing the evaluation of intracellular infection and antileishmanial activity [39]. BALB/c male mice (12 weeks old, weighing 25–30 g) were obtained from the Animal Facility of the Department of Immunology, Parasitology and Pathology at the State University of Londrina (Londrina, PR, Brazil). Animals were maintained under standard laboratory conditions in polyethylene cages (41 × 34 cm), housing up to 15 mice per cage, at a controlled temperature of 22 ± 2 °C and a 12 h light/dark cycle. Mice had free access to sterile water and commercial chow ad libitum. All experimental procedures were conducted in accordance with institucional guidelines and approved by the Animal Experimentation Ethics Committee of the State University of Londrina under the protocol n^o^ 055/2025.

The experimental unit consisted of individual wells containing pooled peritoneal macrophages obtained from BALB/c mice.

#### 2.4.2. Viability of Peritoneal Macrophages

The cytotoxic effects of curcumin (CUR), curcumin-loaded nanoparticles (NP CUR), meglumine antimoniate (AM; 50 µM), antimoniate-loaded nanoparticles (NP AM), the combined formulation (NP CUR + AM), and blank nanoparticles (NP-B) on BALB/c peritoneal macrophages were evaluated using the MTT assay (3-(4,5-dimethylthiazol-2-yl)-2,5-diphenyltetrazolium bromide; Sigma-Aldrich) assay, which measures mitochondrial metabolic activity, as described by Mosmann [40].

Macrophages (5 × 10^5^ cells/mL) were harvested from the peritoneal cavity of BALB/c mice (*n* = 3 per experiment) using ice-cold PBS supplemented with 3% FBS. Cells were pooled to minimize inter-animal variability and cultured in 24-well plates containing 500 µL of RPMI 1640 medium supplemented with 10% FBS for 24 h at 37 °C in a humidified atmosphere containing 5% CO_2_ to allow cell adhesion. Adherent macrophages were then treated with CUR and nanoparticle formulations at concentrations of 12.5, 25, and 50 µM for 24 h under the same conditions. Subsequently, cells were washed with PBS, and MTT solution (0.33 mg/mL) was added, followed by incubation for 4 h. The resulting formazan crystals were solubilized with 300 µL of dimethyl sulfoxide (DMSO), transferred to 96-well plates, and absorbance was measured at 550 nm using a microplate reader (Thermo Scientific, Multiskan GO, Waltham, MA, USA). Untreated macrophages were used as the negative control, whereas macrophages treated with H_2_O_2_ were used as the positive control. The results were expressed as percentage of viable cells compared to the control group and calculated using the following formula: % (viable macrophages) = sample of treated group/average of untreated control) × 100. Each independent experiment was performed using a new pool of animals.

#### 2.4.3. Antiamastigote Assay

BALB/c peritoneal macrophages (5 × 10^5^ cells/mL) were obtained from mice (*n* = 3 per experiment), pooled to minimize inter-animal variability, and seeded onto 13 mm glass coverslips placed in 24-well plates containing 500 µL of RPMI 1640 medium. Cells were cultured for 24 h at 37 °C in a humidified atmosphere containing 5% CO_2_. Adherent macrophages were then infected with promastigote forms of *L. amazonensis* (2.5 × 10^6^ parasites/mL) for 3 h. After infection, non-internalized parasites were removed by washing with PBS, and infected macrophages were treated with CUR, AM, NP CUR, NP AM, or NP CUR + AM at concentrations of 12.5 and 25 µM. RPMI 1640 medium was used as the negative control, blank nanoparticles (NP-B) as the nanoparticle control, and ethanol as the vehicle control. Treatments were carried out for 24 h at 37 °C and 5% CO_2_. Following treatment, cells were fixed and stained with Leishman stain. At least 20 random fields per coverslip were analyzed under oil immersion (1000× magnification) using a light microscope (Olympus BX41, Olympus Optical Co., Ltd., Tokyo, Japan) to determine the infection index and the number of intracellular amastigotes. Each independent experiment was performed using a new pool of animals. Procedures were performed in accordance with Assolini et al. (2020) [34].

#### 2.4.4. Determination of Nitrite as an Estimate of NO Levels in Amastigote Forms

Nitric oxide (NO) production was indirectly quantified by measuring nitrite levels in culture supernatants using the Griess method, as previously described by Gonçalves et al. (2018) [41]. After 24 h of treatment in the antiamastigote assay, 60 µL of culture supernatant was mixed with 60 µL of Griess reagent (1% sulfanilamide and 0.1% *N*-(1-naphthyl)ethylenediamine dihydrochloride in 5% phosphoric acid) and incubated for 10 min at room temperature. Nitrite concentrations were determined using a standard curve generated with serial dilutions of sodium nitrite (NaNO_2_), and absorbance was measured at 550 nm using a microplate reader (Thermo Scientific, Multiskan GO).

#### 2.4.5. ROS Generation in Infected Macrophages

ROS generation in infected macrophages was evaluated as described by Bortoleti et al. (2018) [42]. Macrophages infected with *L. amazonensis* (5 × 10^5^ cells/mL) and treated under the same conditions as in the antiamastigote assay were washed with PBS (pH 7.4) and incubated with the cell-permeant probe 2′,7′-dichlorodihydrofluorescein diacetate (H_2_DCFDA; 2 µM diluted in DMSO) for 30 min at 37 °C in the dark. H_2_O_2_ was used as the positive control, and RPMI 1640 medium served as the negative control. ROS generation was quantified fluorometrically using excitation and emission wavelengths of 488 and 530 nm, respectively.

### 2.5. Statistical Analysis

Data from three independent experiments performed in triplicate were expressed as mean ± standard error of the mean (SEM). Statistical analyses were performed using GraphPad Prism^®^ software (GraphPad Software Inc., San Diego, CA, USA). Data normality was assumed based on the experimental design. Differences among multiple experimental groups were analyzed using one-way analysis of variance (ANOVA), which is appropriate for comparing more than two treatments within the same experimental design. When significant differences were detected, Tukey’s post hoc test was applied to identify pairwise differences between groups, as it provides appropriate control for multiple comparisons in balanced designs. Differences were considered statistically significant at *p* < 0.05.

For assays involving primary macrophages (MTT, antiamastigote, and ROS assays), the sample size (*n* = 3 animals per experiment) was determined based on previous studies and experimental feasibility. No a priori sample size calculation was performed. Macrophages were pooled prior to plating, and each independent experiment was conducted using a new pool of animals. For all experiments, data were obtained from triplicate wells and independently repeated to ensure reproducibility.

## 3. Results and Discussion

### 3.1. Free CUR Reduces the Proliferation of L. amazonensis

**Figure 1:**
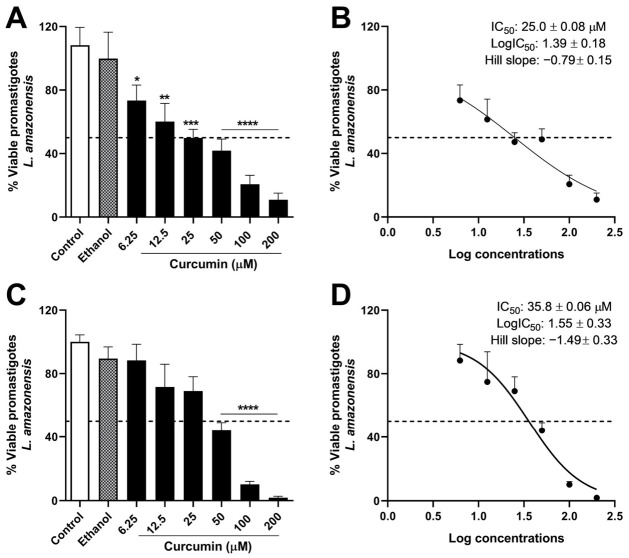
CUR exhibited leishmanicidal activity against *L. amazonensis* promastigotes. Promastigotes were treated with different concentrations of CUR (6.25, 12.5, 25, 50, 100, and 200 µM) or M199 medium (control), and parasite proliferation was analyzed after 24 and 48 h ((**A**) and (**C**), respectively). The concentrations that inhibited 50% of promastigote proliferation at 24 (**B**) and 48 h (**D**) were calculated based on the proliferation assay. The dotted horizontal line represents the 50% viability threshold used to calculate the IC_50_ values from the dose-response curves. Values represent the mean ± SEM of three independent experiments carried out in triplicate. * Significant difference compared to the control (*p* ˂ 0.05), ** (*p* ≤ 0.001), *** (*p* ≤ 0.0005), and **** (*p* ≤ 0.0001).

Initially, the effect of CUR on the proliferation of *L. amazonensis* promastigote forms was evaluated. After 24 h of treatment, all tested concentrations (6.25, 12.5, 25, 50, 100, and 200 µM) significantly reduced parasite proliferation compared with the control group (*p* ˂ 0.05) (Figure 1A). However, after 48 h, only concentrations of 50, 100 and 200 µM promoted a significant reduction in parasite growth (*p* ≤ 0.0001) (Figure 1C). Based on these data, the IC_50_ values were calculated as 25.00 ± 0.08 µM and 35.80 ± 0.06 µM for 24 and 48 h, respectively (Figure 1B,C). The higher IC_50_ value observed after 48 h suggests that CUR activity was not time dependent under our experimental conditions, possibly due to reduced CUR stability or bioavailability during prolonged incubation. Since no time-dependent effect was observed, subsequent experiments were performed using the lowest effective concentration, 25 µM (9.21 µg/mL), after 24 h of treatment.

Several studies have reported the direct leishmanicidal activity of CUR against different *Leishmania* species, including *L. major*, *L. braziliensis*, *L. tropica*, *L. infantum*, *L. donovani*, and *L. amazonensis* [22,25,43,44,45,46]. Das et al. evaluated the activity of CUR against *L. donovani* promastigotes and reported an IC_50_ of 25 µM [21], which is consistent with our results. For *L. amazonensis*, Alonso et al. reported IC_50_ values ranging from 20.2 to 81.9 µM under different parasite densities after 24 h of exposure [18]. Additionally, Spíndola et al. demonstrated that CUR isolated from *C. longa* exhibited an IC_50_ of 14.9 μM, showing superior activity and the highest selectivity index (12.7) compared with plant extracts [47]. Similarly, Gomes et al. reported an IC_50_ of 24.4 µM against *L. amazonensis* promastigotes after 24 h [24], supporting our findings.

### 3.2. Mitochondrial Hyperpolarization and Oxidative Stress

**Figure 2:**
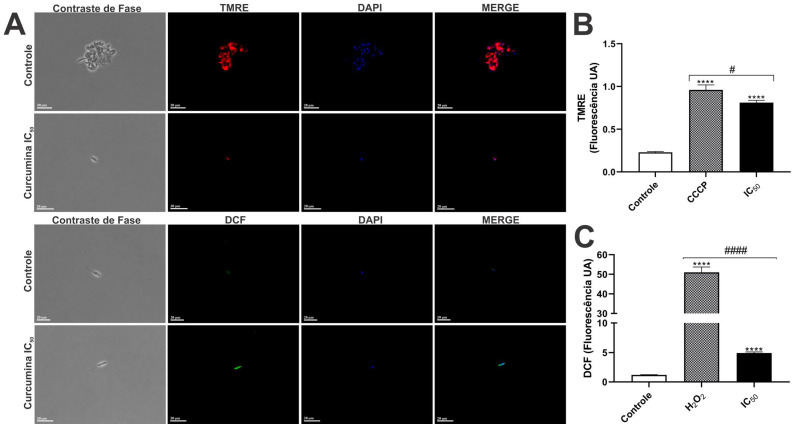
CUR induces mitochondrial hyperpolarization and ROS production in *L. amazonensis* promastigotes. Parasites were treated for 24 h with the IC50 (25 µM) and analyzed for mitochondrial membrane potential using the TMRE assay by fluorescence microscopy and fluorimetry (**A**,**B**), and for ROS production using the H_2_DCFDA probe (**A**,**C**). M199 medium was used as a negative control. Carbonyl cyanide 3-chlorophenylhydrazone (CCCP) and H_2_O_2_ were used as positive controls for TMRE and H_2_DCFDA, respectively. Values represent the mean ± SEM of three independent experiments performed in triplicate. Fluorescence values are expressed as arbitrary units (AU) **** Significant difference compared with the control (*p* ≤ 0.0001). ^#^ Significant difference between treatments (*p* < 0.05), ^####^ (*p* ≤ 0.0001).

Given the leishmanicidal effect of CUR, we investigated the mechanisms involved in promastigote death. The treatment with the IC_50_ of CUR (25 µM) induced mitochondrial hyperpolarization (*p* ≤ 0.0001) (Figure 2B) and increased ROS levels (*p* ≤ 0.0001) (Figure 2C), as evidenced by fluorescence micrographs (Figure 2A). Das et al. reported the dual antioxidant and pro-oxidant roles of CUR, which depend on the dose and environmental conditions [21,48]. At higher concentrations, CUR exhibits pro-oxidant activity due to excessive ROS generation that surpasses its antioxidant capacity [21,49]. In *L. donovani*, an oxidative burst was observed after 1 h of CUR treatment, and pretreatment with N-acetylcysteine (NAC) blocked ROS generation and apoptosis, suggesting that ROS play a central role in CUR-induced programmed cell death (PCD) [21].

*Leishmania* spp. possess a single, highly developed mitochondrion that is essential for parasite survival and energy metabolism [50]. Mitochondria are the primary source of ROS in trypanosomatids, even under physiological conditions, and excessive ROS can cross mitochondrial membranes, disrupt biosynthetic pathways, and induce cytotoxicity [51]. Cao et al. demonstrated that CUR increases ROS generation, leading to mitochondrial DNA (mtDNA) damage and impairment of the electron transport chain, thereby disrupting the mitochondrial membrane potential (ΔΨm) and amplifying oxidative stress [52]. Similarly, treatment with the curcumin monocetone analog (DBA) caused severe mitochondrial damage in parasites [53]. Importantly, mitochondrial hyperpolarization has been proposed as a critical early event in CUR-induced apoptosis [52].

### 3.3. Free CUR Alters Metabolism in L. amazonensis Promastigotes

**Figure 3:**
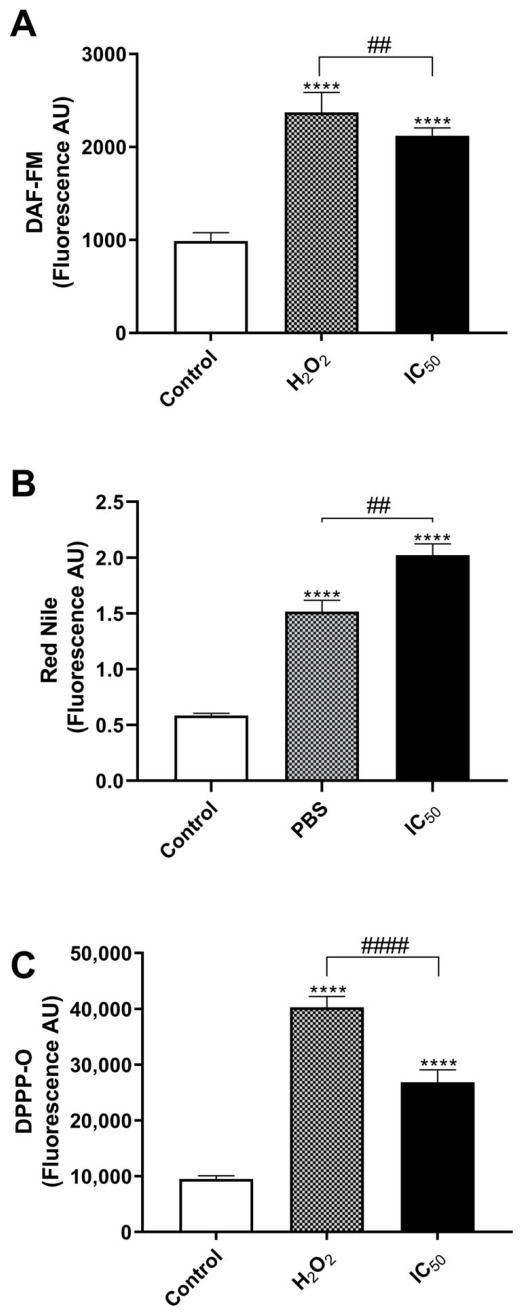
Detection of nitric oxide production, lipid bodies, and lipid peroxidation in *L. amazonensis* promastigotes. Parasites were treated for 24 h with the IC_50_ (25 µM) and analyzed using DAF-FM diacetate for nitric oxide detection (**A**), Nile red staining for lipid bodies (**B**), and diphenyl-1-pyrenylphosphine (DPPP-O) for lipid peroxidation (**C**). M199 medium was used as a negative control. H_2_O_2_ and PBS were used as positive controls for DAF-FM, DPPP-O, and Nile red staining. Values represent the mean ± SEM of three independent experiments performed in triplicate. Fluorescence values are expressed as arbitrary units (AU) **** Significant difference compared with the control. ^##^ Significant difference between treatments (*p* ≤ 0.001). ^####^ (*p* ≤ 0.0001).

ROS-mediated stress affects essential cellular processes, including signaling, metabolism, growth, and apoptosis [54]. In this context, CUR treatment significantly increased NO levels in promastigotes after 24 h (*p* ≤ 0.0001) (Figure 3A). Beyond its microbicidal role, NO acts as a modulator of mitochondrial metabolism and can disrupt key mitochondrial functions, potentially contributing to mitochondrial hyperpolarization [35,55].

Exacerbated oxidative stress promotes damage to proteins, nucleic acids, and lipids [54]. Since oxidative stress is known to induce lipid accumulation in the cytosol as a stress response [56,57], we evaluated lipid droplet formation. CUR-treated promastigotes exhibited a significant accumulation of cytoplasmic lipid droplets compared with the control group (*p* ≤ 0.0001) (Figure 3B). This accumulation is considered a protective mechanism against external stressors [58].

Membrane damage is another hallmark of oxidative stress and can lead to lipid peroxidation [59]. Accordingly, CUR treatment significantly increased lipid peroxidation in *L. amazonensis* promastigotes (*p* ≤ 0.0001) (Figure 3C). Lipid peroxidation directly damages membrane phospholipids and can also act as a signaling mechanism that triggers programmed cell death [59].

### 3.4. Free CUR Induces Death in L. amazonensis Promastigotes

**Figure 4:**
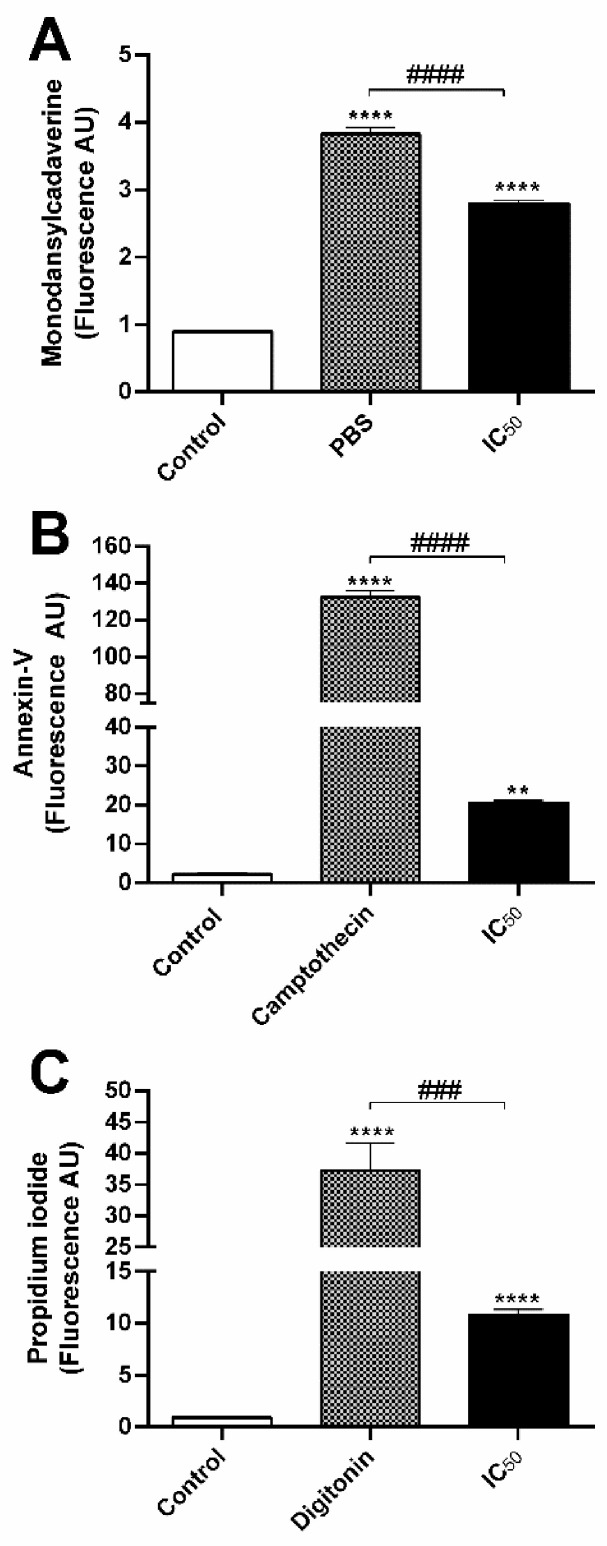
Accumulation of autophagic vacuoles and apoptotic and necrotic processes induced by CUR in promastigote forms of *L. amazonensis*. Parasites were treated for 24 h with the IC50 (25 µM) and analyzed for autophagic vacuoles using monodansylcadaverine (MDC) staining (**A**), phosphatidylserine externalization using annexin V (**B**), and plasma membrane integrity using propidium iodide (PI) staining (**C**) by fluorimetry. M199 was used as a negative control. PBS, Camptothecin, and Digitonin were used as positive controls for MDC, annexin V, and PI assays, respectively. Values represent the mean ± SEM of three independent experiments carried out in triplicate. Fluorescence values are expressed as arbitrary units (AU). ** Significant difference compared to control (*p* ≤ 0.001), **** (*p* ≤ 0.0001). Significant difference between the treatments, ^###^ (*p* ≤ 0.0005) and ^####^ (*p* ≤ 0.0001).

Next, we sought to identify the type of cell death induced by CUR. Treatment with the IC_50_ concentration for 24 h resulted in a significant accumulation of autophagic vacuoles (*p* ≤ 0.0001) (Figure 4A). In *Leishmania*, autophagy plays a crucial role in maintaining cellular homeostasis, enabling survival during nutrient deprivation and participating in differentiation throughout the parasite life cycle [60]. Singh and Chauhan reported that treatment with DBA induced extensive autophagic vacuolization, culminating in autophagic cell death in *L. donovani* promastigotes [61]. Moreover, autophagy-related proteins may contribute to apoptosis or necrosis, depending on the cellular context [62].

Consistent with these findings, CUR treatment significantly increased phosphatidylserine exposure compared with the control group (*p* ≤ 0.001) (Figure 4B). Phosphatidylserine externalization is a classical apoptotic marker in trypanosomatids subjected to stress [63]. Several studies have documented the pro-apoptotic effects of CUR in *Leishmania* spp. [22,53,64]. Basmaciyan et al. demonstrated that CUR induces overexpression of the *LmjF.36.6540* gene, which encodes a conserved hypothetical protein associated with essential cellular processes, and that its dysregulation correlates with increased cell death in *L. major*. Additionally, CUR-induced ROS generation increases cytosolic Ca^2+^ levels in *L. donovani* promastigotes, leading to mitochondrial uncoupling and apoptotic cell death [21].

Alongside apoptosis-like features, increased propidium iodide (PI) uptake was also observed (*p* ≤ 0.0001) (Figure 4C), indicating compromised plasma membrane integrity. According to Basmaciyan and Casanova, *Leishmania* cell death is initially characterized by reduced viability, followed by loss of membrane integrity. To characterize apoptotic cell death, at least two markers must be present, such as DNA fragmentation, cell rounding, cell shrinkage, plasma membrane alterations, and mitochondrial dysfunction. However, late apoptotic cells are often indistinguishable from necrotic cells [65,66].

### 3.5. Free CUR Induces Cell Cycle Arrest and DNA Fragmentation

**Figure 5:**
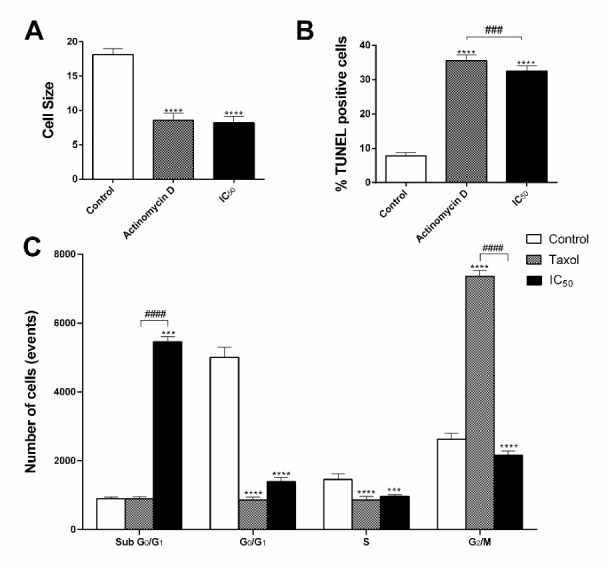
Free CUR reduces cell size and induces DNA fragmentation and cell cycle arrest at the sub-G_0_/G_1_ phase in *L. amazonensis* promastigotes. Parasites were treated for 24 h with the IC_50_ of CUR (25 µM) and analyzed by flow cytometry for cell volume (**A**), DNA fragmentation using the TUNEL assay (**B**), and cell cycle distribution using propidium iodide (PI) staining (**C**). M199 medium was used as a negative control. Actinomycin D and Taxol were used as positive controls for cell size, TUNEL, and cell cycle analyses. Values represent the mean ± SEM of three independent experiments performed in triplicate. *** Significant difference compared with the control (*p* < 0.0001). **** (*p* < 0.0001). ^###^ Significant difference between treatments (*p* < 0.0001). ^####^ (*p* < 0.0001).

Given the antiproliferative and pro-apoptotic effects of CUR, we evaluated cell cycle progression and DNA integrity. Treatment with the IC_50_ concentration significantly reduced promastigote size (*p* ≤ 0.0001) (Figure 5A) and induced DNA fragmentation (*p* ≤ 0.0001) in *L. amazonensis* (Figure 5B). Marcolino et al. demonstrated that CUR combined with photodynamic therapy induces high ROS levels, morphological alterations, disruption of ΔΨm, and causes DNA fragmentation in *L. braziliensis* and *L. major* promastigotes [67,68].

CUR exhibits affinity for DNA [69] and has been detected in both the nucleus and kinetoplast of *Leishmania* parasites [45]. Accordingly, CUR treatment induced a significant accumulation of cells in the sub-G_0_/G_1_ phase (*p* < 0.0001) (Figure 5C), indicative of apoptotic DNA loss. Similar results were reported by Özlem Çalışkan et al., who observed sub-G_1_ accumulation following CUR-associated sonodynamic therapy in *L. tropica* promastigotes [70]. Apoptotic cells are commonly identified by reduced DNA content (sub-G_1_) and characteristic morphological alterations [71].

### 3.6. Free CUR Induces Morphological and Ultrastructural Changes in L. amazonensis Promastigotes

**Figure 6:**
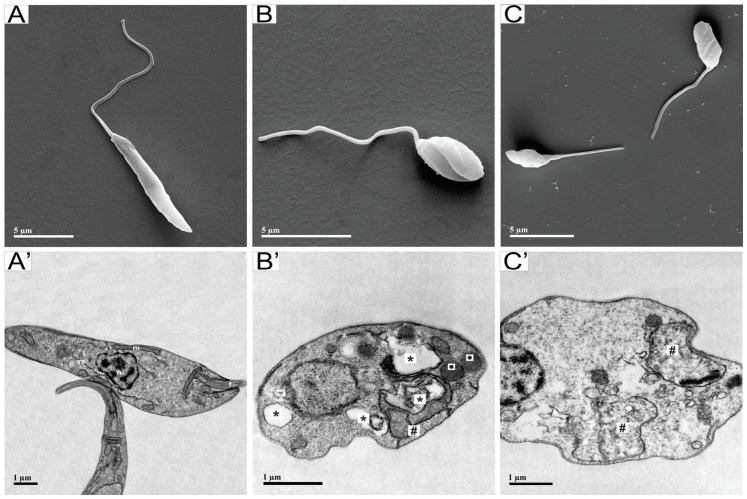
Morphological and ultrastructural alerations in *L. amazonensis* promastigotes induced by CUR. Promastigote forms were treated with CUR (25 µM) for 24 h and analyzed by scanning and transmission electron microscopy. (**A**–**C**) Scanning electron microscopy. (**A’**–**C’**) Transmission electron microscopy. (**A**,**A’**) Untreated *L. amazonensis* promastigotes; (**B**,**C**,**B’**,**C’**) promastigotes treated with CUR IC_50_. Scale bars: 5 µm (**A**–**C**) and 1 µm **(A’**–**C’**). White arrows indicate reduced cell volume; arrowheads indicate flagellar shortening. (**A**) f, flagellum; c, kinetoplast; m, mitochondria; n, nucleus; re, endoplasmic reticulum. (**A’**–**C’**) ●, autophagic vacuoles; * lipid storage bodies; #, mitochondrial swelling.

Scanning (SEM; Figure 6A–C) and transmission (TEM, Figure 6A’–C’) electron microscopy were performed to assess CUR-induced morphological and ultrastructural changes in *L. amazonensis* promastigotes. Untreated promastigotes exhibited typical elongated morphology, flagella proportional to body length, a smooth and intact cell surface, and well-preserved intracellular structures (Figure 6A,A’). In contrast, CUR-treated promastigotes showed flagellar shortening and reduced cell volume, consistent with previous observations (Figure 6B,C). TEM analysis revealed swollen mitochondria, accumulation of autophagic vacuoles and lipid droplets in the cytoplasm of the parasites, as well as DNA fragmentation, confirming the alterations previously observed (Figure 6B’,C’). Despite PI labeling, SEM images do not show membrane rupture or cytoplasmic leakage, suggesting late apoptotic features rather than primary necrosis.

### 3.7. Nanoparticles Reduce the Cytotoxicity of CUR

**Figure 7:**
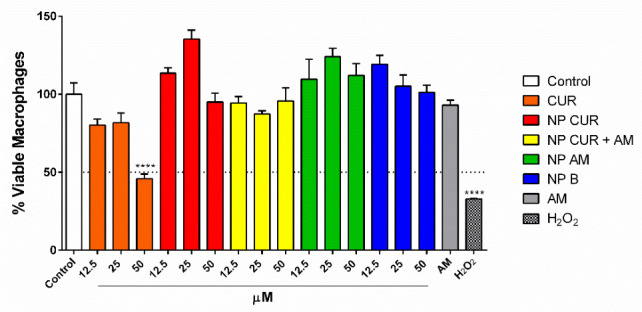
Viability of murine peritoneal macrophages following treatment with free and nanoparticulate formulations. Peritoneal macrophages from BALB/c mice were treated for 24 h with free curcumin (CUR), curcumin-loaded nanoparticles (NP CUR), antimoniate-loaded nanoparticles (NP AM), NP CUR + AM, or blank nanoparticles (NP-B) at concentrations of 12.5, 25, and 50 µM. RPMI medium was used as a negative control. Antimoniate (AM, 50 µM) and H_2_O_2_ were used as positive controls. The dotted horizontal line represents the 50% viability threshold. Values represent the mean ± SEM of three independent experiments performed in triplicate. **** Significant difference compared with the control (*p* < 0.0001).

After establishing the antipromastigote activity of CUR, its cytotoxicity toward BALB/c mouse peritoneal macrophages was evaluated. CUR concentrations of 12.5 and 25 µM were non-toxic, whereas 50 µM induced significant cytotoxicity compared with the control group (*p* ≤ 0.0001) (Figure 7). Spíndola et al. reported a CC_50_ value of 69.75 µg/mL for CUR in RAW 264.7 macrophages [46]. CUR toxicity has been associated with oxidative DNA damage in several cell types [52,72,73,74], and its poor bioavailability further limits its therapeutic application [27].

To address these limitations, targeted drug delivery systems, based on nanoparticle technology, have emerged as a prominent solution [27]. Nanoparticle-based drug delivery systems offer controlled release, improved stability, enhanced loading of hydrophilic and lipophilic compounds, reduced toxicity, as well as high biocompatibility and large-scale production [34,75,76]. In this study, nanoencapsulation completely abolished CUR-induced cytotoxicity at all tested concentrations (12.5–50 µM) for NP CUR, NP CUR + AM, NP AM, and NP-B (Figure 7). Similar reductions in cytotoxicity were reported by Chaubey et al. using mannose-conjugated curcumin-loaded chitosan nanoparticles in J774.1 macrophages [77]. Likewise, Assolini et al. reported that drug delivery systems allow the optimization of pharmacokinetic properties, overcoming common disadvantages of drugs such as low solubility, low permeability, and enzymatic and hydrolytic degradation. This improved efficiency may reduce toxic effects by decreasing the dosage required for therapeutic activity [78]. These findings support the selection of 12.5 and 25 µM for subsequent experiments.

### 3.8. Free CUR and Its Nanoparticles Eliminate Intracellular Amastigotes

**Figure 8:**
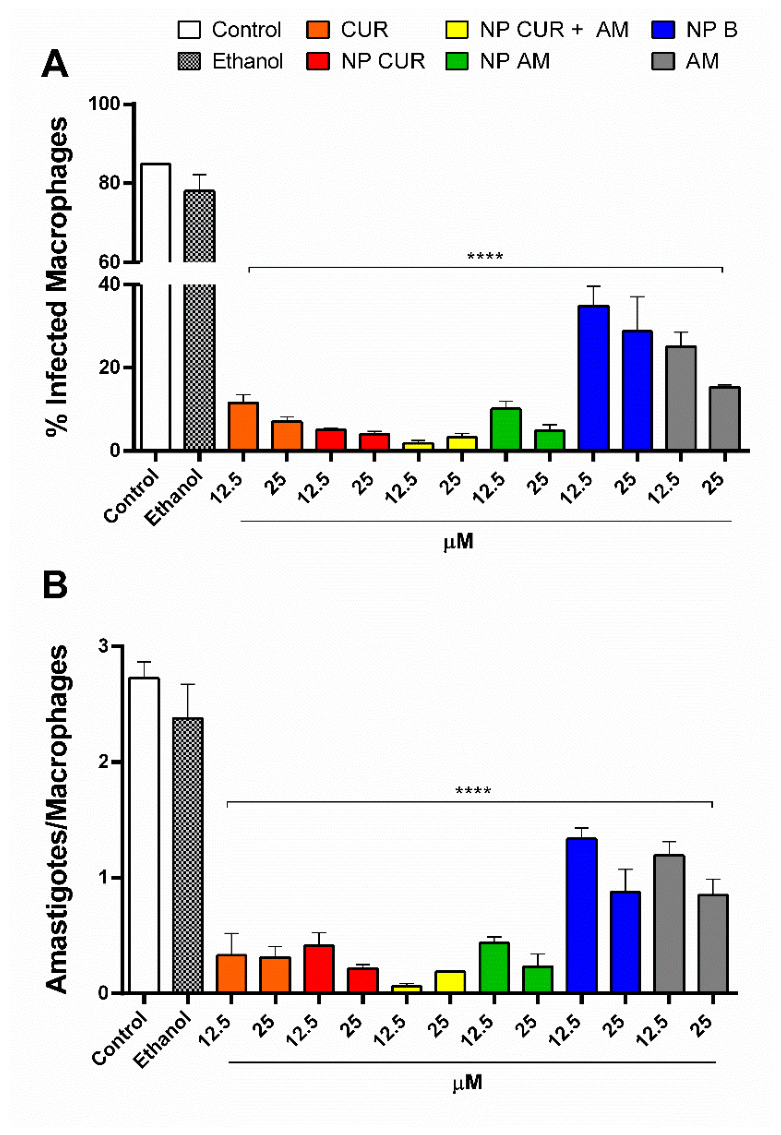
Effects of free and nanoparticulate CUR on *L. amazonensis*-infected macrophages. Peritoneal macrophages infected with *L. amazonensis* were treated with CUR, NP CUR, NP CUR + AM, NP AM, NP-B, or AM at concentrations of 12.5 and 25 µM for 24 h. The percentage of infected macrophages (**A**) and the number of amastigotes per macrophage (**B**) were determined. Values represent the mean ± SEM of three independent experiments performed in duplicate. **** Significant difference compared with the control (*p* < 0.0001).

Macrophages are the primary host cells for *Leishmania*, and the ability of amastigotes to establish themselves within parasitophorous vacuoles is a key determinant of disease pathogenesis [77,79]. Treatment with free or nanoparticle-loaded CUR significantly reduced both the percentage of infected macrophages and the number of intracellular amastigotes at all tested concentrations (*p* ≤ 0.0001) (Figure 8A,B).

Previous studies reported IC_50_ values of 21.12 and 11.77 µM for CUR against *L. major* amastigotes after 12 and 24 h, respectively [19], and 16 µM against axenic *L. mexicana* amastigotes after 24 h [44]. In our study, nanoparticle formulations at 25 µM reduced the number of intracellular amastigotes by 95.9% ± 0.6, 96.7% ± 0.9, and 95.2% ± 1.4 for NP CUR, NP CUR + AM, and NP AM, respectively. Tiwari et al. (2017) also demonstrated the enhanced efficacy of Eudragit-coated nanocurcumin (CNP) formulations, particularly when combined with miltefosine, which exhibited a synergistic effects in J774.1 macrophages infected with *L. donovani* [80].

### 3.9. Mechanisms of Amastigote Elimination

**Figure 9:**
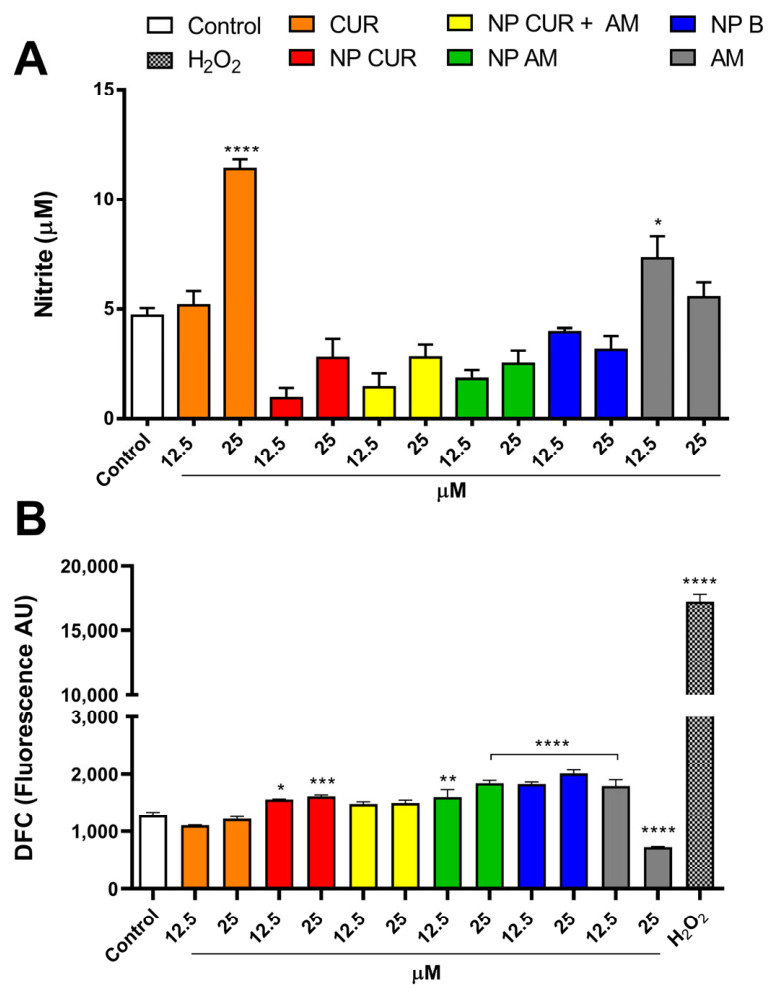
Effects of free and nanoparticulate CUR on NO and ROS production in infected macrophages. Peritoneal macrophages infected with *L. amazonensis* were treated with CUR or nanoparticulate formulations at concentrations of 12.5 and 25 µM for 24 h under the same conditions used for the anti-amastigote assay. Culture supernatants were collected for nitric oxide quantification using the Griess method (**A**). Intracellular ROS production was measured using the H_2_DCFDA probe (**B**). Values represent the mean ± SEM of two independent experiments performed in triplicate. Fluorescence values are expressed as arbitrary units (AU) * Significant difference compared with the control (*p* < 0.05), ** (*p* < 0.01), *** (*p* < 0.001), and **** (*p* < 0.0001).

To elucidate the mechanisms involved in amastigote elimination, NO and ROS production were assessed. Treatment with CUR and AM significantly increased NO levels compared with the control group (*p* < 0.05) (Figure 9A). ROS production increased following treatment with NP CUR, NP AM, NP-B, and free AM at 12.5 µM, whereas AM at 25 µM reduced ROS levels (*p* < 0.05) (Figure 9B).

Endogenous NO production via inducible nitric oxide synthase (iNOS) and ROS generation are central effector mechanism in macrophage-mediated pathogen clearance [20]. Several studies have demonstrated that CUR exerts both antioxidant and pro-oxidant effects, which vary according to concentration, exposure time, and cellular context [81,82,83,84]. Kunwar et al. reported that CUR treatment (1–25 µM) in RAW 264.7 macrophages led to a dose-dependent increase in DCF fluorescence after 2 h; however, after 18 h, ROS levels declined below basal values, indicating that CUR-induced pro-oxidant activity is transient. Similarly, Lee et al. demonstrated that CUR (10 µM) significantly attenuated NO and ROS production in RAW 264.7 macrophages after 24 h by suppressing nuclear factor kappa B (NF-κB) signaling and activating nuclear factor erythroid 2–related factor 2 (Nrf2).

Comparable findings have been reported for nanoparticle-based formulations. Curcumin-loaded nanoparticles and liposomal curcumin effectively inhibited NO production in both alveolar macrophages and RAW 264.7 cells. Nanoparticles, particularly beeswax-based formulations, possess intrinsic antioxidant and anti-inflammatory properties [85], suggesting that nanoparticle-mediated parasite elimination may occur independently of oxidative burst activation.

Collectively, our findings suggest that free CUR eliminates both promastigote and amastigote forms of *L. amazonensis* through the induction of ROS and NO, ultimately triggering autophagic and apoptotic pathways. In contrast, the mechanisms underlying nanoparticle-mediated elimination of amastigote forms remain to be further elucidated, particularly regarding ROS production, immunomodulatory activity, antioxidant response, and iron metabolism.

## 4. Conclusions

Altogether, our results demonstrate that CUR exhibits potent leishmanicidal activity against *L. amazonensis* promastigote forms by promoting ROS generation and triggering autophagic and apoptotic pathways. In infected macrophages, both free CUR and its nanoparticle formulations effectively eliminated intracellular amastigotes, with NO production induced by free CUR contributing to parasite killing. Notably, this study provides the first in vitro evidence elucidating the mechanisms underlying *L. amazonensis* elimination following treatment with CUR, its nanoparticle formulations, and their combinations, highlighting the therapeutic potential of these strategies. In addition, the biocompatibility and biological activity of beeswax-based nanoparticles support their potential future application as topical formulations for cutaneous leishmaniasis.

## Data Availability

The original contributions presented in this study are included in the article. Further inquiries can be directed to the corresponding authors.

## Abbreviations

CUR—curcumin; ROS—reactive oxygen species; AM—antimoniate; NO—nitric oxide; SLNs—solid lipid nanoparticles; O/W—oil/water; W/O/W—water/oil/water; FBS—fetal bovine serum; ∆Ѱm—mitochondrial membrane potential; TMRE—tetramethyl rhodamine ethyl ester; H_2_DCFDA—2′,7′–dichlorofluorescein diacetate; H_2_O_2_—hydrogen peroxide; DCF—2′,7′-dichlorofluorescein; NR—Nile red; DPPP-O—diphenyl-1-pyrenylphosphine; MDC—monodansylcadaverine; CPT—camptothecin; PI—propidium iodide; SEM—scanning electron microscopy; CO_2—_carbon dioxide; TEM—transmission electron microscopy; FSC-H—forward light scatter; NP—nanoparticle; MTT—3-(4,5-dimethylthiazol-2-yl)-2,5-diphenyltetrazolium bromide; DMSO—dimethylsulfoxide; mtDNA—mitochondrial DNA; DBA—synthetic monocetone analog of curcumin; NAC—N-acetylcysteine; PCD—programmed cell death; DMCH—(2E,6E)-2,6-bis (2,3 -dimethoxybenzylidine) cyclohexanone; iNOS—nitric oxide synthase; NF-κB—nuclear factor kappa B; Nrf2—nuclear factor erythroid-2-related factor 2.
